# Detection of Immunity Gap before Measles Outbreak, Ho Chi Minh City, Vietnam, 2024

**DOI:** 10.3201/eid3110.250234

**Published:** 2025-10

**Authors:** Thinh Ong, Cao Thu Thuy, Nguyen Hong Tam, Le Hong Nga, Nguyen Thi Vy Uyen, Truong Thi Thanh Lan, An Tran, Le Thi Kieu Diem, Huynh Phuc Khang, Dang Thi Xuan Dung, Lu Nhat Chuong, Nguyen Hoai Thao Tam, Vu Chau Giang, Tran Thi Bich Cam, Huynh Thi Ngoc Anh, Pham Thi Minh Thu, Dinh Nguyen Huy Man, Nguyen Thanh Dung, Nguyen Le Nhu Tung, Huynh Hong Phat, Le Mau Toan, Nguyen Thanh Lam, Pham Nguyen The Nguyen, Hoang Ngoc Nhung, Tran Tan Thanh, Nguyen Van Vinh Chau, Tang Chi Thuong, Guy Thwaites, C. Louise Thwaites, Marc Choisy, Le Van Tan

**Affiliations:** Oxford University Clinical Research Unit, Ho Chi Minh City, Vietnam (T. Ong, C.T. Thuy, N.T. Lam, P.N.T. Nguyen, H.N. Nhung, T.T. Thanh, G. Thwaites, C.L. Thwaites, M. Choisy, L.V. Tan); Ho Chi Minh City Center for Disease Control, Ho Chi Minh City (N.H. Tam, L.H. Nga, N.T.V. Uyen, T.T.T. Lan, A. Tran, L.T.K. Diem, H.P. Khang, D.T.X. Dung, L.N. Chuong, N.H.T. Tam, V.C. Giang, T.T.B. Cam, H.T.N. Anh, P.T.M. Thu); Hospital for Tropical Diseases, Ho Chi Minh City (D.N.H. Man, N.T. Dung, N.L.N. Tung, H.H. Phat, L.M. Toan); Department of Health, Ho Chi Minh City (N.V.V. Chau, T.C. Thuong); Centre for Tropical Medicine and Global Health, University of Oxford, Oxford, United Kingdom (G. Thwaites, C.L. Thwaites, M. Choisy, L.V. Tan)

**Keywords:** Measles, viruses, disease outbreaks, serology, population surveillance, epidemiology, Vietnam

## Abstract

In 2022, we established a residual sample serosurveillance program in Ho Chi Minh City, Vietnam. During September 2022–April 2024, we found low measles antibody seroprevalence in children in the city’s western region, where a measles outbreak began in May 2024. Serosurveillance could be a useful tool for outbreak prediction and prevention.

Measles is a highly contagious vaccine-preventable disease. Ho Chi Minh City (HCMC), Vietnam, a city of 11 million persons, experienced a 20-month vaccination disruption during COVID-19 lockdowns and mass COVID-19 vaccination campaigns (June−November 2021) and postpandemic vaccine shortages (October 2022−December 2023). The last major measles outbreak in HCMC occurred in 2019. In 2024, measles cases increased in May, leading to an official outbreak declaration on August 27, 2024, in which 4,133 cases were reported by December 2024. Case-patients had a median age of 5.1 years (interquartile range 9 months to 12.6 years; range 1 month to 76 years). HCMC began a measles vaccination campaign on August 31, 2024, targeting children 1–5 years of age, and expanding to children 1–10 years of age on October 1, 2024.

Serosurveillance of antibody titers can be used to assess population immunity and relies on repeated serosurveys to monitor immunity changes over time (*1*). In HCMC, we established a serosurveillance system using anonymized residual serum samples from hospitals, cataloged and stored for future use. Sampling followed an age-stratified serial cross-sectional scheme every 3 to 4 months at Children’s Hospital 1, Children’s Hospital 2, and City Children’s Hospital. The only public pediatric hospitals in HCMC, those hospitals handle most hospitalized children 0–15 years of age across HCMC.

We analyzed 1,097 serum samples from children 0–15 years of age collected during September 2022−April 2024 by using a measles IgG ELISA assay (SERION Immunologics, https://www.serion-immunologics.com). We defined seropositivity as antibody titer >200 mIU/mL (sensitivity 99.0%; specificity 95.0%). We used generalized additive models with a binomial error distribution, logit link, and thin plate spline smooth terms to model seroprevalences as functions of age or time. For time analysis, the models applied population weights to each age. We corrected all models for sensitivity and specificity using the Rogan-Gladen estimate (*2*). We used line list data of patients residing in HCMC to investigate the outbreak. We estimated time-varying reproduction numbers, using previously described methods (*3*), with gamma-distributed serial intervals (mean 14.5 days; SD 3.25 days) (*4*) (Appendix).

During September 2022−April 2024, seroprevalence of measles antibodies among children <15 years of age remained consistently <90% across all 3 hospitals (Figure 1). Such low seroprevalences in the 0–5-year age group, likely resulting from vaccination disruptions, translates to high risk for a measles outbreak. Seroprevalence was lower in the 5–15-year age group (Figure 1), possibly because of incomplete vaccination in the past. The western part of HCMC, corresponding to City Children’s Hospital catchment area (Figure 1), had the lowest seroprevalence and reported the first outbreak cases, highest cumulative incidence rates, and consistently high attack rates compared with other districts (Appendix Figure 1, panels A, C). Seroprevalence remained below the administrative coverage estimates for children who received both first and second measles vaccine doses (Figure 2; Appendix Figure 2, Tables 1, 2).

**Figure 1:**
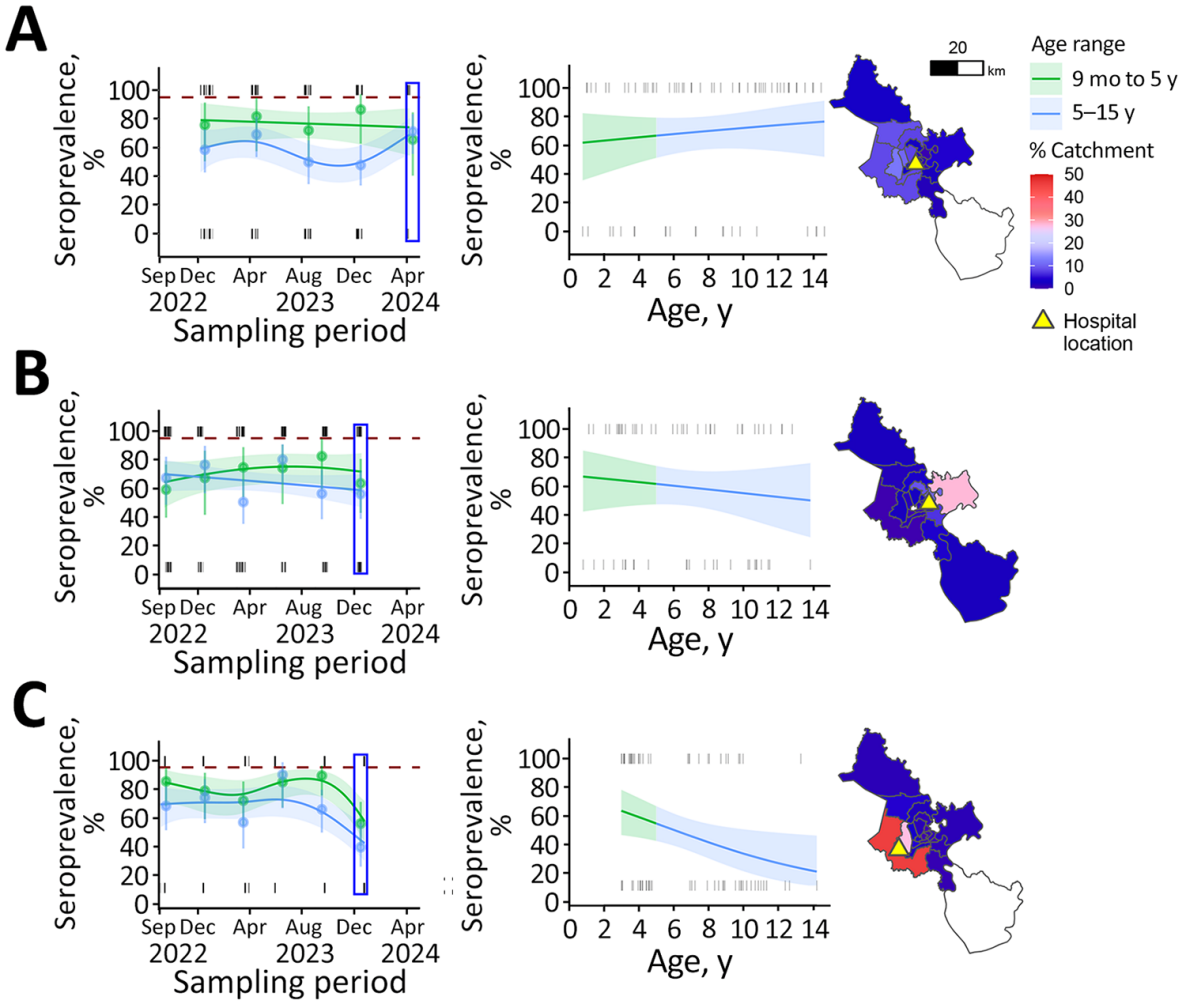
Hospital locations, catchment areas, and number of cases per age in detection of immunity gap before a measles outbreak, Ho Chi Minh City, Vietnam, 2024. A) Children’s Hospital 1; B) Children’s Hospital 2; C) City Children’s Hospital. The first column shows seroprevalence as a function of time of collection for 2 age bands from each hospital; blue rectangles indicate the latest collections; dashed red lines represent the 95% critical vaccination threshold for measles; tick marks at top and bottom of graphs denote seropositive and negative samples; points represent monthly aggregated seroprevalence; error bars indicate 95% CI of the aggregated seroprevalence; shaded areas indicate 95% CI of the seroprevalence estimates by time of collection. Before the measles outbreak, the levels of seroprevalences were 70.7% (95% CI 57.6–81.1) for Children’s Hospital 1 in April 2024 (A), 58.1% (95% CI 44.1–70.9) for Children’s Hospital 2 in December 2023 (B), and 41.5% (95% CI 29.4–54.1) for City Children’s Hospital in December 2023 (C). The middle column shows age-stratified seroprevalence at the most recent timepoint (represented in blue rectangles from the first column). The third column maps the catchment areas based on sample addresses.

**Figure 2:**
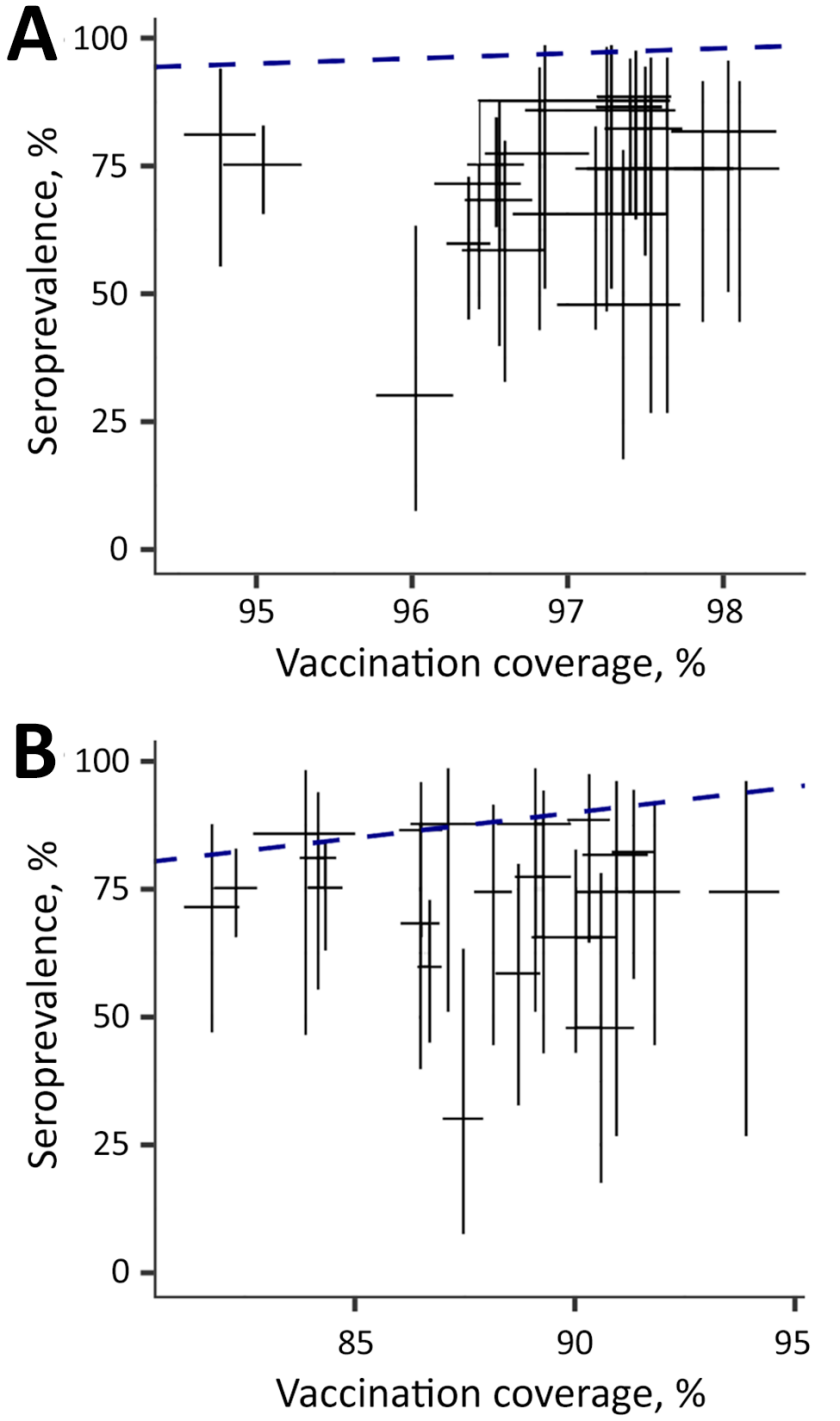
Comparison of seroprevalence and vaccination coverage in a study of immunity gap before a measles outbreak, Ho Chi Minh City, Vietnam, 2024. A) Seroprevalence among children receiving first measles vaccine dose only; B) seroprevalence among children receiving first and second measles vaccine doses. Bars show the 95% CI of seroprevalence (vertical) and vaccine coverage (horizontal). Seroprevalence remained below the administrative vaccination coverage estimates for children who received both first and second measles doses but corresponded more closely with second-dose coverage. The blue dashed line indicates 1:1 relationship of vaccination coverage to seroprevalence. A wider view, administrative coverage data for each district and neighboring provinces, and antibody concentration of 17 children <9 months of age is available in the Appendix.

Reproduction numbers peaked at 1.99 (95% CI 1.65–2.36) on August 13, 2024, and declined to 0.97–1.50 after the vaccination campaign (Appendix Figure 1). The percentage of infected patients 1–10 years of age decreased after the vaccination campaign targeting that group, and the percentage of infected 10–15-year-olds increased, and attack rates rapidly rose. Children 10–15 years of age and adults were infected later than other groups, but their attack rates increased quicker than for other age groups.

Measles antibody seroprevalence gaps might have served as an early warning for this measles outbreak. The lowest seroprevalence, observed in the city’s western region, corresponded to the earliest cases and highest attack rates. Low seroprevalence in persons 5–15 years of age might explain the number of measles cases in that group. Because that age group is the most socially connected in the country’s population (*5*), low immunity can accelerate community spread. Of note, researchers studying a 2014 measles outbreak in northern Vietnam recommended serosurveillance to assess outbreak risks and improve responses (*6*). Strengthening serosurveillance to detect immunity gaps early could enhance preparedness for future outbreaks.

The first limitation of our study is that hospital-based samples might underestimate community immunity. However, small pockets of susceptibility pose high outbreak risks, especially in communities with high average vaccination coverages (*7*). Estimating coverage from registry data is challenging because of variables such as uncertain residency and unrecorded migration from areas with lower vaccination coverage. Our hospital-based serosurveillance system is a valuable example of sentinel surveillance, especially where hospital-acquired infections have triggered previous outbreaks (*8*). A key advantage is efficiency and sustainability in sample collection. Second, although measles antibody titers correlate with protection, limited data are available to establish a protective threshold (*9*). We used the manufacturer-recommended cutoff of >200 mIU/mL to classify seropositivity.

In summary, our results suggest that low-cost, hospital-based serosurveillance data could detect risk for measles outbreaks >6 months in advance. Acknowledging that this approach to surveillance should be further validated to assess the specificity of such a system (false-positive alarms), we are confident that such an approach could effectively identify populations at high outbreak risk, potentially informing optimal vaccination strategies.

Appendix Additional information for detection of immunity gap before measles outbreak, Ho Chi Minh City, Vietnam, 2024.
