# Supplementary material for: Detection of Immunity Gap before Measles Outbreak, Ho Chi Minh City, Vietnam, 2024
Source: Emerg Infect Dis. 2025 Oct;31(10):2059–62. doi: 10.3201/eid3110.250234 (PMC12483019; doi:10.3201/eid3110.250234)
Supplement: Appendix — Additional information for detection of immunity gap before measles outbreak, Ho Chi Minh City, Vietnam, 2024. [file 25-0234-Techapp-s1.pdf]

Article DOI: http://doi.org/10.3201/eid3110.250234

EID cannot ensure accessibility for supplementary materials supplied by authors. Readers who have difficulty accessing supplementary content should contact the authors for assistance.

Detection of Immunity Gap before Measles Outbreak, Ho Chi Minh City, Vietnam, 2024

Appendix

Methods

Measles cases data

Measles cases were defined as all outpatients and inpatients diagnosed with measles at hospitals or clinics across Ho Chi Minh City, reported to the national infectious diseases surveillance system database in accordance with the Laws on Prevention of Infectious Diseases.

Residential addresses of patients were retrieved from the national infectious diseases surveillance system database. Our dataset was limited to patients whose residential addresses were within Ho Chi Minh City. The date of symptom onset was recorded as the first day the patient exhibited rash symptoms. Patient age was calculated by subtracting the date of birth from the date of symptom onset.

Vaccination records data

Vaccination records were extracted from Vietnam's National Immunisation Information System, an electronic registry launched in 2017 that reached stable operation in 2018. Our dataset was limited to children whose registered residence is Ho Chi Minh City. Each child is assigned a unique vaccination identification number at birth. When the recorded residence address is Ho Chi Minh City, the child is followed by the city's Centre for Disease Control (HCDC). For every dose administered, the registry stores the date of vaccination and the corresponding vaccine antigen. Data on 2023 measles vaccination coverage in the 19 southern provinces of Vietnam were obtained from the Expanded Program on Immunization Summary Report 2023, published by the National Institute of Hygiene and Epidemiology, Ministry of Health.

Serum bank

Samples were obtained from two serum banks that routinely collect residual blood samples from hospitals based on serial cross-sectional sampling. The OUCRU serum bank, established in 2009, collects serial cross-sectional samples every 4 months (April, August, and December) from 10 hospitals in southern Vietnam, with five samples per 1-year age group from 0 to 15 years, resulting in a total of 75 samples per collection timepoint (*1*). Samples from Children's Hospital 1 were collected following the OUCRU serum bank protocol.

The HCDC serum bank, established in 2023, collects serial cross-sectional samples every 3 months (March, June, September, and December) from 15 hospitals in Ho Chi Minh City since 2022. Samples from Children's Hospital 2 and City Children's Hospital were collected according to the HCDC serum bank protocol. For each timepoint, 35 samples were collected from children aged 0–5 years, 35 samples from those aged 5–12 years, and 5 samples from those aged 12–15 years, making a total of 75 samples per collection timepoint.

All samples were collected within 1 month at each collection timepoint. Blood samples are collected by venipuncture as part of routine care. A residual volume of serum was collected and stored at the serum bank after biochemistry/hematology tests have been conducted at the laboratories as part of standard out- and in-patient clinical assessment and care.

Inclusion criteria

- Residual serum samples from biochemistry/hematology laboratories of collaborating hospitals.
- Samples of patients reside in the province where the collaborating hospital located.
- Samples of patients from non-infectious diseases departments.
- Minimum sample volume is 0.2ml.
- Samples have recorded information about exact day of birth, gender, commune of residence, collection day and ward origin.
- The vials containing samples must be intact with tight cap. The color of samples is not cloudy when visualizing by eyes.

Exclusion criteria

• Samples missing required information in inclusion criteria.

• Samples from infectious disease departments.

• Samples of patients who reside outside the province where the hospital is located.

• Samples are known to be a duplication (from the same patient) of a sample submitted to this study.

• Sample container is leaked or turbid samples.

Catchment areas of the three hospitals

The catchment areas of the three hospitals were calculated at the district level as the percentage of samples from each district, determined by the address of residence, divided by the total number of samples.

Measles assay

Measles IgG ELISA assay (SERION Immunologics, Würzburg, Germany) was used. Following the manufacturer's recommendation, seropositivity was computed using the >200 mIU/mL antibody titer threshold, associated with a sensitivity of 99.0% and specificity of 95.0%.

Seroprevalence modeling

A generalized additive model with a logit link and thin plate spline smooth terms for time of collection and age was applied to model seroprevalence using the mgcv R package (*2*), with optimal degree of smoothing was determined by restricted maximum likelihood. When calculating seroprevalence for the population over time (first panels of Figure 1), the model was weighted by the population size of each age group based on 2019 census data. Seroprevalence and its confidence interval were corrected for sensitivity and specificity using Rogan-Gladen estimate (*3*).

$$\pi_{true} = \frac{\pi_{est} + SP - 1}{SE + SP - 1}$$

In which

• π_{true} is the true prevalence with confidence interval (c, d)

• π_{est} is the raw estimated with confidence interval (a, b)

• SE is sensitivity

• SP is specificity

Confidence interval of the true prevalence (c, d) is estimated by:

$$c = \max\left(0, \frac{a + SP - 1}{SE + SP - 1}\right)$$

$$d = \min\left(1, \frac{b + SP - 1}{SE + SP - 1}\right)$$

Quantitative antibody titers of children under 9 months old were modeled as a function of age using a generalized additive model with a gamma distribution and a log link function.

Vaccination coverage

In Vietnam, the first measles vaccine dose is recommended at 9 months of age, and the second dose at 18 months. Data were available for children born from 2018 to 2023 to calculate:

• First-dose coverage among children aged 9 months to 5 years

• Second-dose coverage among children aged 18 months to 5 years

Only children whose registered residence is Ho Chi Minh City were available in the dataset. Coverage was calculated as:

$$\text{Coverage}_{\text{1st}} = \frac{\text{Number of children (9m} - \text{5y) who received a first measles dose}}{\text{Total number of children (9m} - \text{5y) residing in HCMC}}$$

$$\text{Coverage}_{\text{2nd}} = \frac{\text{Number of children (18m} - \text{5y) who received a second measles dose}}{\text{Total number of children (18m} - \text{5y) residing in HCMC}}$$

Attack rate

Attack rates (AR) at day t were computed as:

$$AR_t = \frac{Cases_t}{Population\ size - \sum_{i=1}^{t-1} Cases_i}$$

Attack rates by district were computed using the population size of each district and cases reported with residential addresses within that district. Similarly, attack rates by age group were calculated using the population size of each age group. Population size data were retrieved from

the latest Ho Chi Minh City census conducted in 2019. Daily attack rates were smoothed using a 7-day moving average with a 1-day step.

Results

In the western districts of the city, administrative data report first-dose measles coverage of 95.0%–96.5%, reaching the critical herd-immunity threshold to avoid a measles outbreak, while the second dose coverage falls to 82.3%–84.3% (Appendix Table 1, Appendix Figure 2). Official records showed that Ho Chi Minh City has the highest measles coverage in the region at 96.8%, with most neighboring provinces only around 50%–70% (Appendix Table 2).

Seroprevalence was consistently lower than both first- and second-dose administrative coverage, a pattern also observed during the 2014 outbreak (*4*). In practice, estimating coverage from administrative data (when available) faces several challenges. Coverage is calculated by dividing the number of vaccinated children by the total number of children living in the city. While the numerator is available from registry data, the denominator (the total number of resident children) is much harder to determine accurately.

Several factors contribute to this uncertainty. Many children live in the city temporarily (e.g., for school) but are officially registered elsewhere, and thus excluded from city records. Internal migration is common, but families moving from other provinces often do not update their household registration. Moreover, age information in the vaccination registry is not always reliable. Ho Chi Minh City is known for high rates of population movement, yet registration updates are neither mandatory nor automatically linked to the immunisation registry. As a result, the actual number of children eligible for vaccination is unknown, and administrative data likely overestimate coverage. A survey conducted by local CDC found that up to 30% of families with children under five had no official registration and were therefore excluded from administrative estimates (*5*). A study in Vietnam reported that unregistered families are less likely to participate in vaccination programmes due to the lack of household registration (*6*), further supporting concerns about overestimation.

Finally, administrative coverage only reflects vaccination uptake, not actual immunity. Population immunity (seroprevalence) results from both vaccination and natural exposure. This is particularly relevant for older children and adults who may not have been vaccinated due to

historically low coverage but could still have developed immunity through past infections or when, as is the case in Vietnam, the vaccine registry was put in place only recently.

Serosurveillance addresses these limitations in our study by sampling children based on their self-reported residence at hospitals, and measures antibodies from both vaccination and natural exposure. It is reported that hospital-acquired infections have triggered previous measles outbreaks in Vietnam (*7*). By assessing immunity levels among the population visiting hospital, we directly evaluate early signal of outbreak risk of a key potential source of future outbreaks.

References

1. Anh DD, Choisy M, Clapham HE, Cuong HQ, Dung VTV, Duong TN, et al. Plans for Nationwide Serosurveillance Network in Vietnam - Volume 26, Number 1—January 2020 - Emerging Infectious Diseases journal - CDC. [cited 2022 Dec 1]; https://wwwnc.cdc.gov/eid/article/26/1/19-0641_article

2. Wood SN. Fast stable restricted maximum likelihood and marginal likelihood estimation of semiparametric generalized linear models. J R Stat Soc Series B Stat Methodol. 2011;73:3–36. https://doi.org/10.1111/j.1467-9868.2010.00749.x

3. Diggle PJ. Estimating prevalence using an imperfect test. Epidemiol Res Int. 2011;2011:608719. https://doi.org/10.1155/2011/608719

4. Choisy M, Trinh ST, Nguyen TND, Nguyen TH, Mai QL, Pham QT, et al. Sero-Prevalence Surveillance to Predict Vaccine-Preventable Disease Outbreaks; A Lesson from the 2014 Measles Epidemic in Northern Vietnam. Open Forum Infect Dis. 2019;6:ofz030. PubMed https://doi.org/10.1093/ofid/ofz030

5. Department of Infectious Diseases Prevention. Surveillance of vaccination management in Ho Chi Minh City. In: Proceedings of the 2024 Annual Meeting on National Vaccination Programme, Vietnam. 2024.

6. Hoang TA, Oosterhoff P, Le LA, Dinh PN. Equitable Access and Public Attitudes to Vaccination for Internal Migrants in Vietnam [Internet]. The Institute of Development Studies and Partner Organisations; 2023 Feb [cited 2025 May 20]. https://opendocs.ids.ac.uk/articles/report/Equitable_Access_and_Public_Attitudes_to_Vaccination_for_Internal_Migrants_in_Vietnam/26434411/1

7. Tran DM, Ong T, Cao TV, Pham QT, Do H, Phan PH, et al. Hospital-acquired infections and unvaccinated children due to chronic diseases: an investigation of the 2017-2019 measles outbreak in the northern region of Vietnam. BMC Infect Dis. 2024;24:948. PubMed https://doi.org/10.1186/s12879-024-09816-w

Appendix Table 1. Vaccination coverage of districts across Ho Chi Minh City in 2023

Region	District	Coverage 1st dose, %	Coverage 2nd dose, %
West	Binh Chanh	95.0	82.3
West	Binh Tan	96.5	84.3
South	7	97.5	91.3
South	Can Gio	97.6	93.9
South	Nha Be	96.8	89.3
North	12	94.8	84.2
North	Cu Chi	96.0	87.5
North	Hoc Mon	96.6	86.5
East	Thu Duc	96.4	86.7
Central	1	97.2	90.0
Central	10	98.0	90.9
Central	11	96.9	87.1
Central	3	97.5	90.9
Central	4	97.3	89.1
Central	5	97.3	83.9
Central	6	98.1	91.8
Central	8	96.4	81.8
Central	Binh Thanh	96.6	88.7
Central	Go Vap	97.9	88.1
Central	Phu Nhuan	97.4	90.6
Central	Tan Binh	97.4	90.3
Central	Tan Phu	97.4	86.5

Appendix Table 2. Vaccination coverage (first dose) of Ho Chi Minh City and neighboring provinces in 2023, in descending order

Province	Coverage 1st dose, %
Ho Chi Minh City	96.8
Tien Giang	96.1
Binh Duong	93.4
Vinh Long	90.7
Bac Lieu	90.0
Can Tho	89.8
Hau Giang	89.4
Ca Mau	87.8
Soc Trang	87.4
Ba Ria Vung Tau	85.3
Dong Nai	79.4
Tay Ninh	76.2
Kien Giang	74.2
Binh Phuoc	73.7
Ben Tre	72.4
Dong Thap	72.4
Long An	72.1
An Giang	63.6
Tra Vinh	45.4

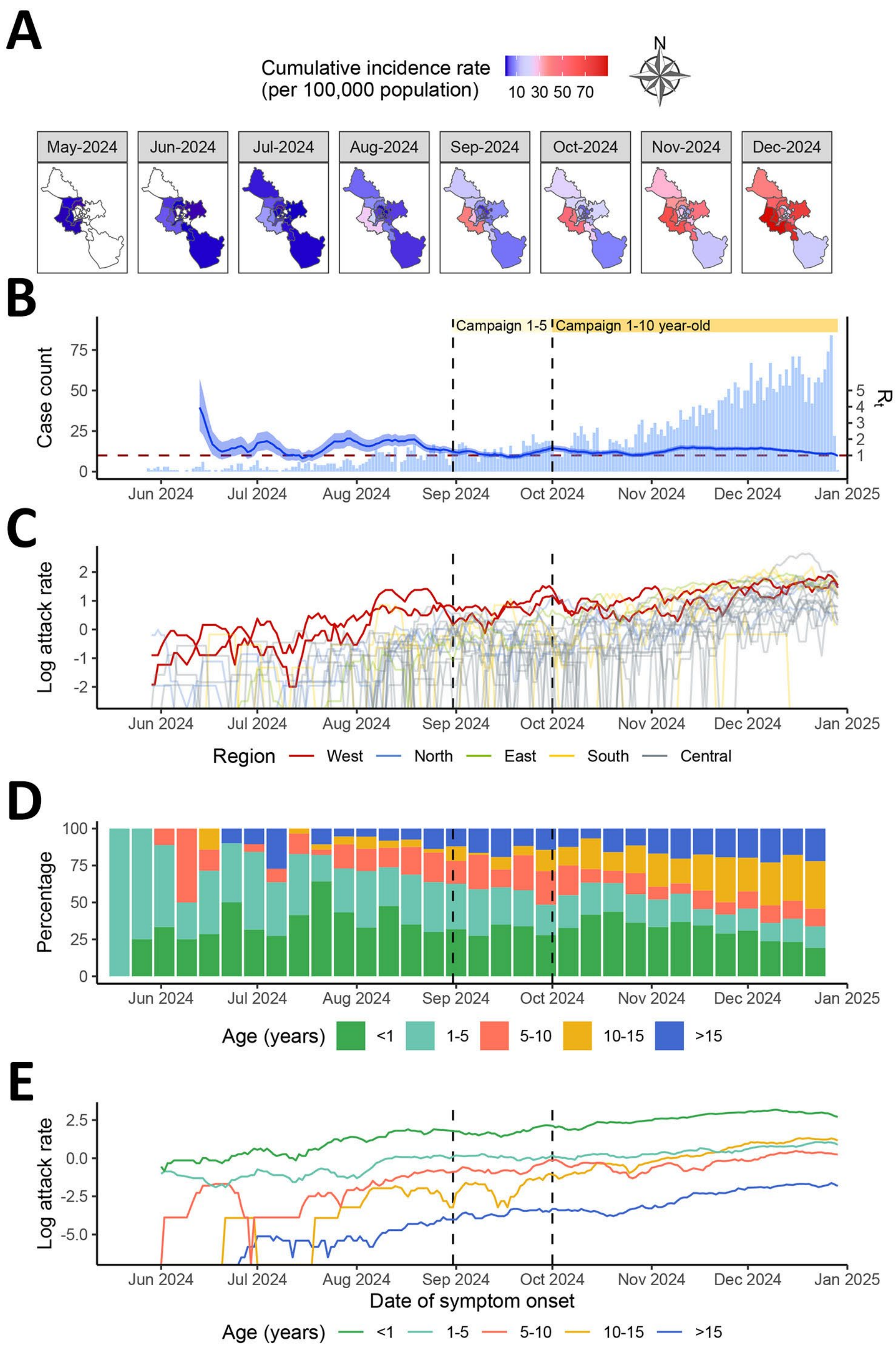

Appendix Figure 1. (A) Spatial distribution of cumulative incidence rate per 100,000 population over time. (B) Daily case count (bars) and instantaneous time-varying reproduction numbers Rt (blue line, with shaded confidence intervals), the dashed red line indicates Rt = 1. (C) Log attack rates per 100,000 population across 22 districts of Ho Chi Minh City, with the two districts on the west side of the city showing the lowest seroprevalence before the outbreak highlighted in red. (D) Weekly distribution of age groups shown as percentages over time. (E) Log attack rates per 100,000 population, stratified by age group.

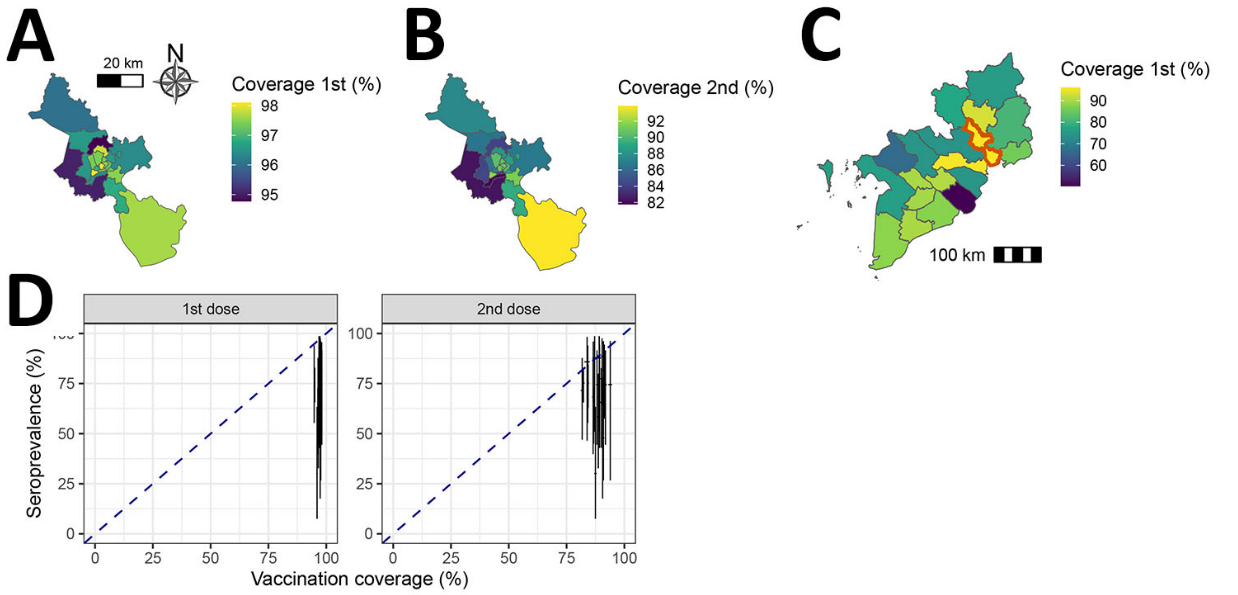

Appendix Figure 2. (A) Map of first-dose administrative measles coverage in Ho Chi Minh City (2023). (B) Map of second-dose administrative measles coverage in Ho Chi Minh City (2023). (C) Administrative first-dose measles coverage in Ho Chi Minh City (red border) and neighboring provinces (2023). (D) Comparison of seroprevalence and administrative vaccination coverage; error bars show 95% confidence intervals, with the dashed blue diagonal line represents the 1:1 relationship.

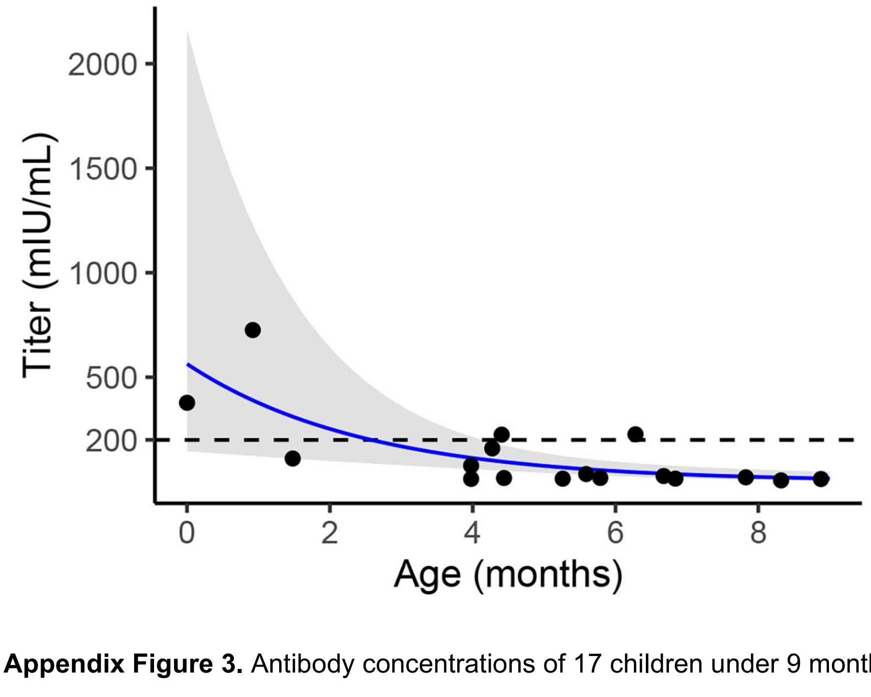

Appendix Figure 3. Antibody concentrations of 17 children under 9 months old, with the blue line representing a fit using a generalized additive model with a gamma distribution of errors, thin plate spline smooth terms and a log link function. The horizontal dashed line indicates the threshold of 200 mIU/mL for seropositivity.

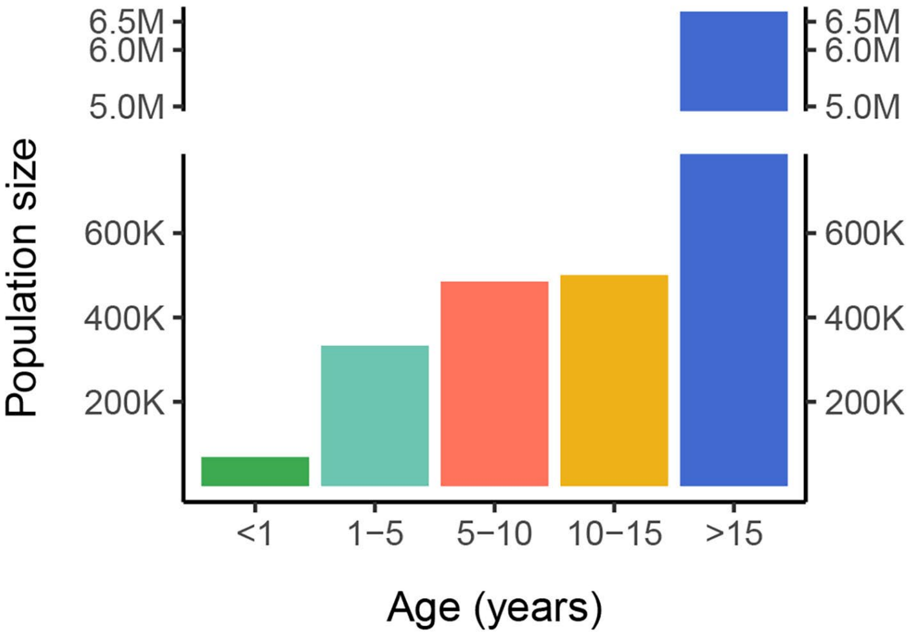

Appendix Figure 4. Population size of age groups in the general population of Ho Chi Minh City, displayed with a broken y-axis.
